# Willin, an Upstream Component of the Hippo Signaling Pathway, Orchestrates Mammalian Peripheral Nerve Fibroblasts

**DOI:** 10.1371/journal.pone.0060028

**Published:** 2013-04-08

**Authors:** Susana Moleirinho, Calum Patrick, Andrew M. Tilston-Lünel, Jennifer R. Higginson, Liselotte Angus, Maciej Antkowiak, Susan C. Barnett, Michael B. Prystowsky, Paul A. Reynolds, Frank J. Gunn-Moore

**Affiliations:** 1 Medical and Biological Sciences Building, School of Medicine, University of St. Andrews, St. Andrews, United Kingdom; 2 Medical and Biological Sciences Building, School of Biology, University of St. Andrews, St. Andrews, United Kingdom; 3 Department of Pathology, Albert Einstein College of Medicine, Bronx, New York, United States of America; 4 Institute of Infection, Immunity and Inflammation, University of Glasgow, Glasgow, United Kingdom; Institute of Molecular and Cell Biology, Singapore

## Abstract

Willin/FRMD6 was first identified in the rat sciatic nerve, which is composed of neurons, Schwann cells, and fibroblasts. Willin is an upstream component of the Hippo signaling pathway, which results in the inactivation of the transcriptional co-activator YAP through Ser127 phosphorylation. This in turn suppresses the expression of genes involved in cell growth, proliferation and cancer development ensuring the control of organ size, cell contact inhibition and apoptosis. Here we show that in the mammalian sciatic nerve, Willin is predominantly expressed in fibroblasts and that Willin expression activates the Hippo signaling cascade and induces YAP translocation from the nucleus to the cytoplasm. In addition within these cells, although it inhibits cellular proliferation, Willin expression induces a quicker directional migration towards scratch closure and an increased expression of factors linked to nerve regeneration. These results show that Willin modulates sciatic nerve fibroblast activity indicating that Willin may have a potential role in the regeneration of the peripheral nervous system.

## Introduction

The Salvador/Warts/Hippo (Hippo) signaling pathway controls tissue growth and organ size by promoting a normal fine tuned homeostasis. Originally characterized in *Drosophila melanogaster*, this pathway limits overgrowth, which may lead to cancer development by inhibiting cell proliferation and promoting apoptosis [1]–[4]. Highly conserved, the *Drosophila* Hippo pathway core components and downstream effectors have direct orthologues in mammals. This functional conservation is confirmed by the ability of mammal orthologues to rescue *Drosophila* mutants *in vivo*; the core components Hpo, Sav, Wts and Mats have respective vertebrate homologues MST1 and MST2, WW45 (also known as Sav1), the kinases LATS1 and LATS2 and MOB1 (MOBKL1A/B). The main targets of the mammalian Hippo signaling cascade are the two *Drosophila* Yki orthologues, Yes-associated protein (YAP) and the transcriptional coactivator with PDZ binding motifs (TAZ). Mimicking the regulation process observed in *Drosophila* Yki when in the presence of high cell densities, LATS phosphorylates YAP at Ser127 and TAZ at Ser89 creating a protein 14-3-3 binding site, which in turn leads to YAP/TAZ cytoplasmic localization. Defects in the Hippo pathway lead to a lack of YAP/TAZ cytoplasmic sequestration which results in YAP/TAZ nuclear accumulation and, eventually, in tumour development [5]–[8] Indeed, we and others have previously described that deregulation of the Hippo signaling cascade components results in YAP dephosphorylation, nuclear sequestration and overgrowth [4], [9]–[15]. Less is known about the upstream components of the core kinase cassette. Merlin, KIBRA (WWC1) and Willin (FRMD6) have been shown to be upstream of MST1/2 and to be able to induce LATS and YAP phosphorylation [9], [11], [16]–[19]. Yet, so far only Merlin is functionally validated *in vivo*, although recently we showed that reduced KIBRA expression in primary breast cancer specimens correlates with the claudin-low subtype [11].

Several lines of research have established a new role for the Hippo signaling pathway in tissue regeneration. Tissue repair, after a major injury, relies on the expansion and/or dedifferentiation of an existing population of progenitor cells, such as primary satellite cells [20] which may recapitulate the developmental process involving the reprogramming of diverse molecular mechanisms [21]. Here, repression of pathways involved in developmental tissue growth, such as the Hippo kinase cascade, induces YAP stemness properties by promoting a controlled cell proliferation aiming restore of organ function [22], [23]. Regeneration of the peripheral nerve is a dynamic process initiated by Schwann cells and the extracellular matrix, which confers a growth promoting environment to peripheral axons. The signal transmitted by damaged axons to Schwann cells informing them of their intention to degenerate is mediated by Raf/MEK/ERK signaling pathway and triggers myelinated Schwann cells to dedifferentiate to a progenitor-like cell [24], [25]. Regrowing axons and Schwann cells migrate towards the nerve gap to promote reinnervation of the distal stump, and relies on signals mediated by both the ephrin/Eph pathway and fibroblasts [26].

Willin (FRMD6) was first identified in the rat sciatic nerve [27] which is composed of neurons, Schwann cells and fibroblasts, and is the nearest human sequence homologue to the *Drosophila* protein Expanded (Ex) sharing 60% of homology with the Ex FERM domain. In *Drosophila*, the lack of Ex expression has been shown to be associated with overgrowth of certain structures such as the wing and imaginal discs [28] reflecting a direct role in controlling cell growth in these tissues. In mammals, recently the FERM domain of Willin was shown to be sufficient to activate the Hippo pathway via MST1/2 and to antagonize YAP-induced phenotypes in mammalian cells [9]. Willin activated the Hippo pathway, inducing the phosphorylation of MST1/2, LATS1 and YAP in MCF10A and 293T cells. Knockdown of Willin expression mimicked YAP overexpression with respect to inducing an Epithelial-Mesenchymal transition (EMT) phenotype in MCF10A cells [9]. As Willin was first identified within the mammalian sciatic nerve, we sought what its functions could be within this structure and whether it shares the same functionalities as in epithelial cells. We show that in mammalian peripheral nerve fibroblasts Willin expression can control the phosphorylation of MST1/2, LATS1 and YAP and thereby YAP’s translocation from the nucleus to the cytoplasm. We also demonstrate that activation of the Hippo pathway is an upstream regulator of ephrinB/EphB signaling and EGFR, which are known factors to be involved in nerve regeneration, and in addition we show that Willin expression can control the rate at which wounds may close. Therefore our results demonstrate that Willin and the Hippo kinase cassette play a role in the maintenance of peripheral nerve system homeostasis.

## Materials and Methods

### Schwann Cells and Fibroblasts Purification

Schwann Cells (SC) were purified using a modification of the method described by Brockes and colleagues [29]. All procedures were carried out in accordance with the guidelines, set forth by the Animals Scientific Procedures Act under schedule 1 procedures. Mice had access to food and water, ad libitum. Furthermore, all procedures were carried out in accordance with the guidelines, set forth by the Animals Scientific Procedures Act, under a project license (No. 6003895) granted by the UK Home Office and with the approval of the University of Glasgow Ethical Review Process Applications Panel. Briefly, sciatic nerve was dissected from neonatal P7 Sprague-Dawley pups. The tissue was minced finely and enzymatically digested with trypsin (100 µl of 2.5%, Invitrogen) and collagenase 500 µl collagenase (1.33%; ICN Pharmaceuticals). The mixed cell population was cultured in 10% FBS without specific SC mitogens for approximately 48 hrs before the addition of cytosine arabinoside (AraC, 10^−5^ M, Sigma, Dorset, UK) for a further 48 hrs to eliminate rapidly dividing fibroblasts. Further purification was then carried out by trypsinising the cells and resuspending them in a small volume of serum free media with anti-Thy1.1 antibody at room temperature for 15 min (1∶50 supernatant, Sigma, Dorset, UK), followed by the addition of rabbit complement (1∶4, Harlan Laboratories Ltd., UK) for 45 min at 37°C [30]. All cell cultures were grown in PLL coated tissue culture flasks. To isolate fibroblast from sciatic nerve a culture was set up as described above but the fibroblasts were not removed by the Thy1.1 complement-mediated kill. The cultures were fed with 10% FBS and the fibroblast over grew the Schwann cells.

### Cell Culture

Purified Schwann cells were grown in Dulbecco’s Modified Eagle Medium (DMEM) supplemented with 10% foetal bovine serum, 2 mM L-glutamine, 100 mg/ml streptomycin, 100 units penicillin/ml, 10 µM forskolin and 20 ng/ml heregulin β1 (R&D Systems, Europe, Oxon, UK). Purified fibroblasts were grown in DMEM supplemented with 20% foetal bovine serum, 2 mM L-glutamine, 100 mg/ml streptomycin, 100 units penicillin/ml. Both cell types were grow in a humidified incubator at 37°C with 5% CO_2_. All experiments were carried out between passage 4 and 18.

### Plasmid Construction

The human Willin-HA ORF was cloned into the pBABEpuro vector (pBabe-puro, Addgene, Cambridge, MA, USA) as an BamHI-EcoRI fragment. The human pBABE-YAP1-Flag (Addgene plasmid 15682) expression clone was described previously (Overholtzer et al., 2006; Angus et al., 2012). pBABE constructs were packaged in PhoenixA cells for viral production. Retroviral infection of primary fibroblasts was performed accordingly standard protocols. The transduced cells were selected, after 24hours, with 2.5 µg/mL puromycin or with 300 µg/mL hygromycin. The selections continued for 5 days (puromycin) or 10 days (hygromycin) after which cells were harvested for downstream experiments. siRNA targeting Willin/FRMD6 (ID #s141256) and a non-targeting siRNA control (ID #4390844 (Ambion, Warrington, UK) were transfected at a concentration of 20 nM, using Lipofectamine 2000 (Invitrogen, Paisley, UK), according to the manufacturer’s instructions and as described previously ([7], [9], [11] Moleirinho et al., 2012; Angus et al., 2012; Zhang et al., 2008).

### In situ Hybridization

*In situ* hybridization was performed with cRNA probes on sciatic nerves of 21 day old mice as previously described [31]. Briefly, RNA was prepared by TRIZOL extraction (Life Technologies) for RT-PCR. Reverse transcription was performed with 1 µg of RNA using SuperScriptTMII reverse transcriptase (400 U; Life Technologies) in the presence of random hexamers (Life Technologies). After incubation at 42°C for 45 min, reactions were terminated by heating at 80°C. DNA in 1 µl of the first strand reaction was amplified by PCR with Dynazyme (Flowgen). Primer sequences were selected from the published neurofascin (ankyrin-binding protein) sequence (available from Gen- Bank/EMBL/DDBJ under accession No. L11002) [32], that would specifically amplify product from the 155- splice variant of neurofascin. A probe specific for NF155 was amplified from within the third FNIII domain of neurofascin with a forward primer, 5′-CCTGAACAGCACAGCCATCAG-3′ (nucleotides 3,550–3,570) and a reverse primer 5′-GACCACAACCATCTCCTGCTTG-3′ (nucleotides 3,775–3,754). The products were cloned into the pGEM-T vector (Promega) and sequenced. The cDNA templates were linearized with either NotI or NcoI and transcribed with either T7 (antisense probes) or SP6 RNA (sense probes) polymerases (Boehringer Mannheim), respectively. Probes specific for Willin were made using the plasmid pSPORT-Willin using the T7 promoter for sense and the SP6 promoter for antisense probes. For cloning into pSPORT: sense 5′-ATAAGAATGCGGCCGCTTTCAGGATGTAGTCCTTCC-3′ and anti-sense 5′-TTACGCGTCGACATGCAGGACCGCCGCAGAGTG-3′ were used for PCR using the 163SciII clone (Accession Number: AF441249) (pSPORT-Willin). All constructs were sequenced prior to use.

### RNA Extraction and Quantitative Real-time PCR Detection

Extraction of RNA from cell lysates was performed using peqGold MicroSpin Total RNA kit (peqLab, Sarisbury Green, UK) followed by cDNA synthesis of 1 µg DNase-digested RNA using First Strand cDNA Synthesis kit for RT-PCR (AMV) (Roche, Lewes, UK). Quantitative PCR of the synthesized cDNA was conducted using SYBR Green 2x Master Mix (Agilent, Wokingham, UK), Mx3005P machine (Agilent) and the following primers: Willin, Merlin, ezrin, MST1/2, LATS1/2, KIBRA, YAP, EphrinB2, EGFR, CTGF, BMP2, FGF1, RASSF8, IGFBP3 and PRL. All measurements were conducted in triplicate unless otherwise indicated and standardized to the levels of β-actin. Sequence of the qPCR primer pairs were as follows (5′ to 3′ direction):

Willin – FW: CAGCCCACAACACAATGAAC RV: AGTGCAGCACCTGTTTCCTT

Ezrin – FW: CCCGGCCGATCCCAATTTGTGAA RV: GGCGGAGACACGTCGGGAC

Merlin - FW: TTTGCCATAGGCAGCCCGCC RV:GTTACACCCACCACTCCTCAAATACC

KIBRA – FW: GTGGAGGGGCGAGCAGGAGA RV: TGGCGTTCTGCTTCCAGGCG

MST1 - FW: TGCTTACTTGGTAACCCAGCCTCAG RV: TGGGACTCGGTCCTCAGGGGA

MST2– FW: AGCAGGACTTCAAGAACAAGAGTCATG RV: GGCGGCTTCAGTCGCAGGTT

LATS1– FW: TGCCGCAAAGGCCGAGCATA RV: TGGCATTGATAGGTCTGGCAGCT

LATS2– FW: TGAGCGCAGAGACGGTGGGT RV: ACGTCCAATGTTTTGGCATAGCTGATT

YAP – FW: AGCCCAAGTCCCACTCGCGA RV: ACGAGGGTCAAGCCTTGGGTC

EphrinB2– FW: TCCCTTTGTGAAGCCAAATC RV: GTCTCCTGCGGTACTTGAGC

EGFR – FW: AACCAGTGTGCCGCAGGGTG RV: GTTCAGGCCGACAACCGCCA

CTGF – FW: CTTGCCTGGATGGGGCCGTG RV: TCCCGGGCAGCTTGACCCTT

BMP2– FW: CCCCTTATCCCGGCCTTCGGA RV: TTTGAGCTGGCTGTGGCAGGC

FGF1– FW: GCCATGGACACCGAAGGGCT RV: GCGCAGCCAATGGTCAAGGGAAC

RASSF8– FW: GGCTGCAGACGGGGAAGCTG RV: GCCGGCAGCACAGTGACCTT

IGFBP3– FW: CCAGCACACACCGCGTGACT RV: GTGGACGCCCCTGGGACTCA

PRL – FW: CAGCCAAGTGTCAGCCCGGA RV: GTGTCTGGCAGTCGCCACCA

### Immunoblot Analysis

Cells were lysed in a lysis buffer composed by 10 mM Tris at pH 8.0, 150 mM NaCl, 1% Na Deoxycholate, 1% Nonidet P-40, 1% Sodium Dodecyl Sulfate, 1 mM EDTA, 1∶25 protease inhibitor cocktail and phosphatase inhibitors. Protein lysates (30 µg) were run on an SDS polyacrylamide gel and transferred onto PVDF transfer membrane (GE Healthcare, Amersham, UK). Primary antibodies used as follows: MST1/2, Phospho-MST1 (Thr183)/MST2(Thr180), LATS1, Phospho-LATS1(Ser909), YAP, Phospho-YAP (Ser127), (1∶1000; Rabbit) (Cell Signaling Technology, Hitchin, UK), TAZ/WWTR1 (LS-B94; 1∶200; Rabbit) (LifeSpan Biosciences, Nottingham, UK), EphrinB2 (ab75868; 1∶1000; Rabbit) (Abcam, Cambridge, UK), EGFR (sc-03; 1∶500; Rabbit) (Santa Cruz Biotechnology, CA, USA), Lamin-β (1∶200; Rabbit) (Insight Biotechnology, Middlesex, UK), β-actin (A1978; 1∶10 000; Mouse) (Sigma-Aldrich, Dorset, UK). Willin rabbit polyclonal antibody was generated against the unique sequence KEASKGIDQFGPPMIIHC of Willin (residues 86–102) which was also used to affinity purify the antibody from the resulting serum (1∶1000; Rabbit) (Dundee Cell Products, Dundee, UK). Secondary antibodies used as follows: horseradish peroxidase (HRP)-conjugate goat anti-mouse (#115-035-062) or anti-rabbit (#111-035-045) (1∶10 000) (Stratech, Newmarket, UK). Levels of protein expression were quantified using Image J software and student’s unpaired *t*-test used to test statistical significance.

### Nuclear/Cytoplasmic Fractionation

Primary fibroblasts subcellular fractionation was performed using a modification of the method described by Angus and colleagues [9]. Briefly, after reached the desired confluence cells were harvested, pelleted at 228 g rpm for 4 min, washed with PBS and pelleted again at 228 g for 4 min. Pellet was resuspended in 0.5 ml of ice-cold cytoplasmic extraction buffer (10 mM HEPES pH 7.9, 1.5 mM MgCl_2_, 10 mM KCl, 0.5 mM DTT and cocktail of protease inhibitors) and kept on ice for 5 min. Cells were lysed with 20 strokes using a pre-chilled Dounce homogenizer and the lysate was centrifuged at 228 g for 5 min at 4°C. The pellet (nuclear fraction) was resuspended in 1 ml of ice-cold nuclear extraction buffer (0.25 mM sucrose, 10 mM MgCl_2_ and cocktail of protease inhibitors), layered over a sucrose bed (0.88 mM sucrose, 0.5 mM MgCl_2_ and cocktail of protease inhibitors) and centrifuged for 10 min at 4°C. Pellet was resuspended in 500 µl of 1x RIPA buffer (50 mM Tris pH 7.5, 150 mM NaCl, 1% NP-40, 0,5% sodium deoxycholate and cocktail of protease inhibitors), briefly sonicated on ice and centrifuged at 2800 g for 5 min at 4°C. The supernatant (cytoplasmic fraction) was transferred to a new 1.5 ml tube, 300 µl of 1x RIPA buffer was added and the lysate centrifuged at 2800 g for 10 min at 4°C to pellet any solids. The supernatant was transferred to a new tube. Protein sample buffer was added to both nuclear and cytoplasmic fractions and 30 µg of protein lysates run on an SDS/10% polyacrylamide gel.

### In vitro Wound Healing Assay

For both overexpression and knockdown analysis the same number of cells were plated in each well of a 6-well culture plate (8×10^5^ and 3×10^5^, respectively). For the knockdown experiments, cells at 60% confluence were transfected on two consecutive days with siFRMD6 or siCtr and allowed to grow until reaching confluence (3 days after the initial transfection). Monolayers of confluent cultures were gently scratched with a sterile micropipette tip and the migration towards the wound was monitored for up to 16 h (overexpression) or 40 h (knockdown). Phase-contrast images were captured after the scratch for each one of the time points. The percentage of scratch covered by cells was measured as the percentage of the invaded area with respect to the initial wound area and calculated using Image J software.

### BrdU/PI labeling and Flow Cytometry

For cell cycle progression analysis, cells were cultured until desired confluence and pulse-labeled with 10 µM BrdU (Sigma-Aldrich, Dorset, UK) for 1hour at 37°C. After trypsinization and two PBS/1% BSA washes cells were fixed in 70% ethanol at 4°C for at least 30 min, centrifuged at 500 g for 10 min at 10°C and the pellet was resuspended in 2 N HCl for 30 min on ice for DNA denaturation. Cells were centrifuged at 500 g for 10 min at 10°C and washed with 0.1 M of sodium tetraborate pH 8.5, centrifuged again and stained with anti-BrdU (# 347580, BD Biosciences, Oxford, UK) for 1 hour, room temperature, in the dark. Cells were then washed and stained with Dylight 488-goat anti-mouse IgG (#115-485-062, Stratech, Newmarket, UK) for 1hour at RT. Stained cells were resuspended in PBS containing 20 µg/ml of propidium iodide (Sigma-Aldrich, Dorset, UK) and RNase at 100 µg/ml for 30 min at RT. For cell cycle analysis using propidium iodide, cells were washed twice in ice-cold PBS, fixed in 70% ethanol at 4°C for at least 30 min, washed twice in ice-cold PBS and then resuspended in PBS containing 4 µg/ml of propidium iodide and RNase at 100 µg/ml for 30 min at RT, in the dark. Cells were then analysed on a FACScan (BD Biosciences, Oxford, UK) using CELLQUEST software.

## Results

### Willin is Expressed in Fibroblasts within the Sciatic Nerve

**Figure 1:**
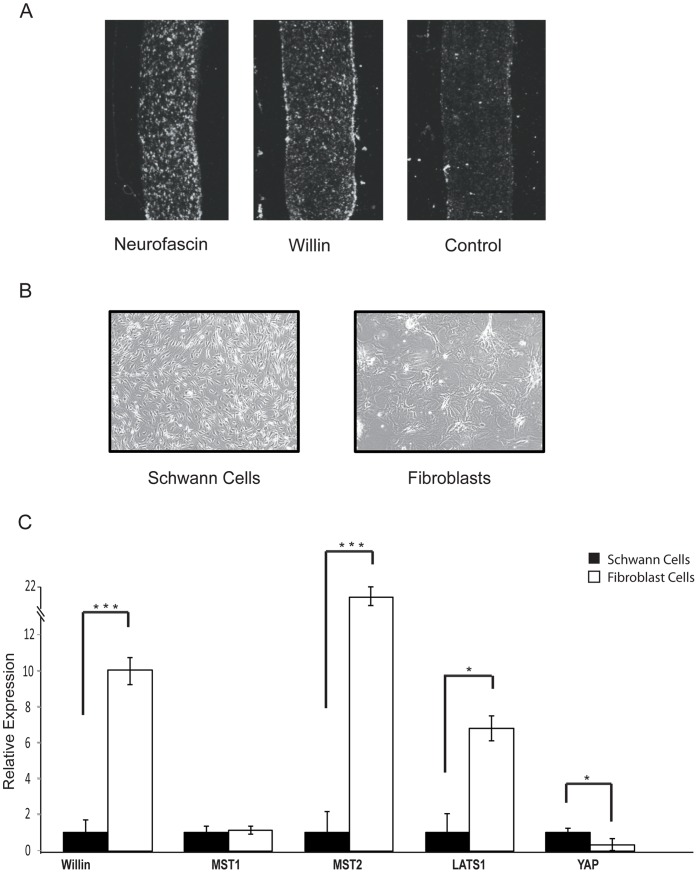
Willin is mainly expressed in primary fibroblasts within the sciatic nerve. (A) Differential expression of Neurofascin 155 and Willin transcripts in rat PNS. Rat sciatic nerve sections were hybridized with riboprobes specific for NF155 or Willin and viewed by dark-field microscope. NF155 transcripts were dispersed around the sciatic nerve, consistent with Schwann cells location at the endoneurium. A robust expression of Willin mRNA was observed at the perineurium suggesting that Willin is expressed in a different population of cells within the sciatic nerve. (B) Pure Schwann cells and fibroblasts cultures isolated from the sciatic nerve present distinct morphologies. Representative phase-contrast images of cells growing in monolayers cultures further confirmed the successful isolation of pure Schwann cells and fibroblasts populations. (C) The hippo signaling pathway is present in the sciatic nerve. mRNA expression of the Hippo pathway components Willin, MST1/2, LATS1 and YAP was determined by quantitative real-time PCR in fibroblasts cells. mRNA levels were compared with Schwann cells (SC set to 1). Willin, MST2, LATS1 mRNA levels are increased in the fibroblasts whereas YAP mRNA expression decreases in these cells. Means were calculated from *Ct* values in three independent experiments. β-actin was used to normalize for variances in input cDNA. Error bars represent ±s.e. (*n* = 3). Schwann cells vs fibroblasts for all the analyzed genes: *p<0.05; **p<0.01; ***p<0.001, Student’s *t*-test. Schwann cells vs fibroblasts: MST1 (p = 0.75); Student’s *t*-test.

Willin was first identified by a yeast two-hybrid screen of a rat sciatic nerve cDNA library using neurofascin as bait and northern blot analysis had confirmed Willin’s expression in this structure [27]. To establish where Willin is located within the sciatic nerve, we compared the mRNA expression pattern of Willin with the Schwann cell expressing isoform of the transmembrane receptor neurofascin [33] by *in situ* hybridization. The Willin transcript appeared to be predominately located at the perineurium (the periphery) of the sciatic nerve (Figure 1A), but also within discrete cells within the endoneurium. However, from their morphology these cells appeared to be different from those in which the glial neurofascin isoform transcript was expressed (Figure 1A). The sciatic nerve is composed of cell bodies of Schwann cells and fibroblasts; therefore, to determine the expression of Willin in either of these cells, both Schwann cells and fibroblasts were cultured from rat sciatic nerve (Figure 1B). From these cultures, quantitative RT-PCR demonstrated that the Willin transcript was expressed 10-fold more in the fibroblasts than in the Schwann cells (Figure 1C). Since Willin is an upstream component of the Hippo pathway [9], [11] components of this newly emerging signaling cascade were also analyzed to determine whether they were expressed within the sciatic nerve. Interestingly, MST2 and LATS1 were more strongly expressed within the fibroblasts than Schwann cells, whilst YAP was less expressed and MST1 was present in both cell types at the same level as assessed by quantitative RT-PCR (Figure 1C).

### Willin Expression Influences the Activation of the Hippo Pathway in Fibroblasts

**Figure 2:**
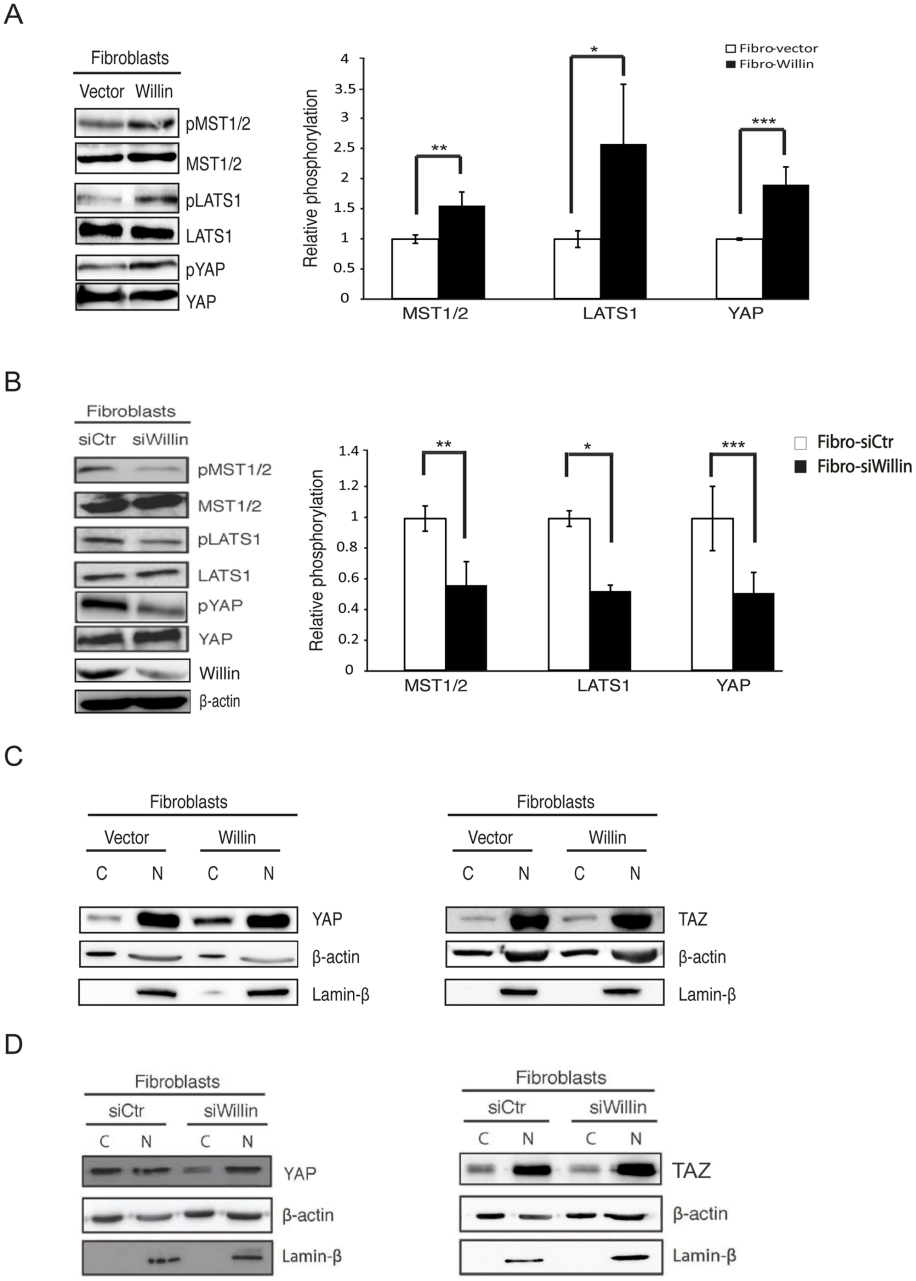
Willin activates the Hippo signaling pathway in fibroblasts of the sciatic nerve. Immunoblot analysis of the retroviral infected fibroblasts with either Willin or empty vector shows (A) Willin increases MST1/2, LATS1 and YAP phosphorylation. Relative phosphorylation to total proteins levels (MST1/2, LATS1 or YAP) are shown and background phosphorylation (in Fibro-vector) is set to 1. Error bars represent ±s.e. (*n* = 3). Fibro-vector vs Fibro-Willin: *p<0.05; **p<0.01; ***p<0.001; Student’s *t*-test. (B) Willin knockdown induces YAP, LATS1 and MST1/2 dephosphorylation. Primary fibroblasts were transfected with either non-targeting siRNA (siCtr) or siRNA targeting Willin (siWillin) for two consecutive days. Cell lysates for western blotting analysis were prepared 48 h post-transfection. The ratios between relative YAP, LATS1 and MST1/2 phosphorylation levels to total proteins (YAP, LATS1 and MST1/2) are shown and background phosphorylation of Fibro-siCtr was arbitrarily set to 1. Immunoblot analysis of efficient Willin knockdown is also presented. β-actin was used as a loading control. Means were calculated from three independent experiments. Error bars represent ± s.e. (*n* = 3). Fibro-siCtr vs Fibro-siWillin: *p<0.05; **p<0.01; ***p<0.001; Student’s *t*-test. (C) Willin expression results in YAP, but not TAZ, translocation from the nucleus to the cytoplasm. Cytoplasmic and nuclear fractions were separated for western blot analysis as indicated. The blots shown are representative of three independent experiments (*n* = 3). (D) Willin knockdown in primary fibroblasts sequesters YAP, but not TAZ, in the nucleus. YAP and TAZ subcellular location was determined by immunoblotting analysis of cytoplasmic and nuclear fractions. β-actin and Lamin-β were used as loading controls for the cytoplasmic and nuclear fractions, respectively. The blots shown are representative of three independent experiments (*n* = 3).

Previously, it has been shown that Willin expression in epithelial MCF10A cells induces YAP phosphorylation at Ser127, by a MST1/2 and MOBKL1A/B-dependent mechanism [9], [11]. Phosphorylation of YAP at Ser127 by LATS1/2 results in YAP translocation to the cytoplasm and then its subsequent ubiquitination and concomitant degradation [4], [8]. Since YAP regulation could be context-dependent, Willin was investigated to determine whether it could activate the Hippo pathway in primary fibroblasts derived from the rat sciatic nerve. Primary fibroblasts were infected with retroviruses expressing either Willin or an empty vector, and stable pools of cells were selected. Upon ectopic Willin expression (Fibro-Willin cells), as predicted there was a significant increase in MST1/2, LATS1 and YAP phosphorylation when compared to the empty-vector control (Fibro-Vector) cells (Figure 2A). These findings were further supported by knocking down endogenous Willin using RNA interference. Down-regulation of Willin using siRNA (Fibro-siWillin cells) caused a significant reduction in MST1/2, LATS1 and YAP phosphorylation (Figure 2B), when compared with control cells (Fibro-siCtr). Knockdown of Willin was confirmed by immunoblotting analysis (Figure 2B). Furthermore, lysates from Fibro-Willin or Fibro-Vector cells were separated into cytoplasmic and nuclear fractions. Efficient phosphorylation of YAP at Ser127 induces cytoplasmic retention through binding to 14-3-3 proteins [8]. As expected, Willin expression promoted YAP sequestration in the cytoplasm. Surprisingly, the localization of TAZ, the paralogue of YAP, was unaffected by Willin expression (Figure 2C). Moreover, investigations were also carried out to determine whether YAP dephosphorylation was accompanied by YAP sequestration in the nucleus upon Willin knockdown. Indeed, YAP nuclear retention was observed upon Willin knockdown (Fibro-siWillin cells) when compared with control cells (Fibro-siCtr). However, TAZ consistently did not translocate from the cytoplasm to the nucleus in this scenario (Figure 2D). Taken together, the data suggest that within sciatic nerve fibroblasts, Willin is an upstream regulator of YAP but not TAZ.

### Willin Suppresses Cellular Proliferation in a Cell-cycle Independent Manner

**Figure 3:**
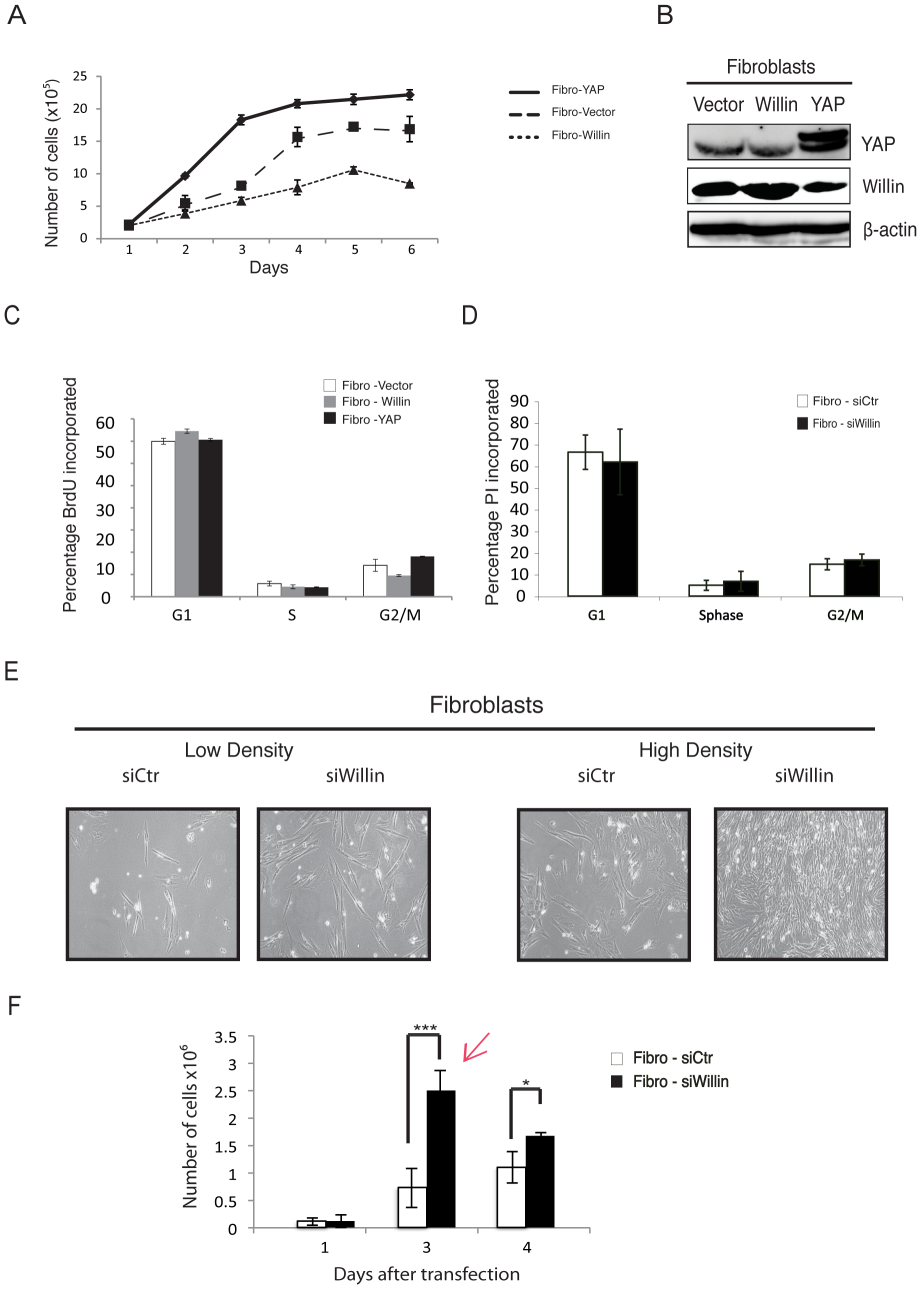
Ectopic expression of Willin, but not YAP, suppresses cell proliferation in the sciatic nerve. Primary fibroblasts were retroviral infected with Willin, YAP or an empty vector. Stable pools were selected and maintained in normal fibroblasts medium. (A) Proliferation curves of fibroblasts stably expressing Willin, YAP or an empty vector, over a 6-day time course, show that Willin suppresses cellular proliferation whereas YAP induces it. (B) Immunoblot analysis of YAP and Willin overexpression in primary fibroblasts. β-actin was used as a loading control. (C) Willin and YAP cell proliferation patterns are cell cycle independent. Willin, YAP and vector-overexpressing fibroblasts were cultured to confluence. Cells at a similar density were pulse-labeled with 10 µM BrdU for 1hour, followed by staining with anti-BrdU and propidium iodide (20 µg/ml for 30 min) for flow cytometry. No statistical significant arrest of cells residing in G0/G1, S or G2/M phases was observed. The mean percentage of cells in the different cell cycle phases was determined. Error bars represent ±s.d. (*n* = 6). (D) Willin knockdown induces cellular proliferation in a cell-cycle independent manner. Cells at a similar density were fixed in 70% ethanol and stained with propidium iodide (4 µg/ml for 30 min) for FACScan analysis. No statistical significant arrest of cells residing in G0/G1, S or G2/M phases was observed. The mean percentage of cells in the different cell cycle phases was determined. Error bars represent ±s.d. (*n* = 6). (E) Representative phase-contrast images of fibroblasts growing in monolayer cultures transfected with either non-targeting siRNA (siCtr) or siRNA targeting Willin (siWillin) for two consecutive days. Images were taken at low and high density. At low density, siWillin fibroblasts present a large and flat spindle-shape with multiple oval nucleoli with extended and interconnected cell processes protruding from the body of each cell. At high density, the cells are confluent and arranged in parallel arrays with the interconnected processes organized in a dense and close network. This effect is not observed in Fibro-siCtr cells. (F) Proliferation curve of control fibroblasts (siCtr) or knockdown Willin (siWillin) cells. Cells were transiently transfected with the respective siRNAs and the growth curve monitored over 4 days. 48 h after the second transfection Fibro-siWillin cells show a 3.46 fold increase in cell proliferation when compared with the Fibro-siCtr cells. Red arrow – experimental time point indicating when cells were harvested for downstream analyses. Each data point is the mean of three independent experiments. Error bars represent ±s.d. (*n* = 3). Fibro-siCtr vs Fibro-siWillin: *p<0.05; ***p<0.001; Student’s *t*-test.

To explore the effect of Willin and YAP expression on cell proliferation, primary fibroblasts were infected with retroviruses expressing Willin, YAP1 (Fibro-YAP) or an empty vector. Stable pools of cells were selected and equal numbers of cells were cultured and counted every day for 6 days (Figure 3A). Fibro-Willin cells showed a significant delay in proliferation when compared to Fibro-YAP or Fibro-Vector cells. Interestingly, ectopic YAP expression resulted in increased cellular proliferation, when compared with Fibro-Vector cells (Figure 3A). Ectopic expression of Willin and YAP was confirmed by immunoblotting analysis (Figure 3B).

To determine if the slower proliferation rate of Fibro-Willin cells was associated with cell cycle changes, these cells were analyzed by flow cytometry. Fibro-Willin, Fibro-YAP or Fibro-Vector cells were seeded at 1×10^6^ cells (corresponding to day 2 of Figure 3A) and 24 h later they were pulse-labeled with BrdU for 1 hour and probed by flow cytometry. There was no statistically significant difference in the number of cells residing in G0/G1, S or G2/M phases (Figure 3C) in any of the cell-types tested, suggesting that there was no cell cycle arrest in Fibro-Willin cells.

Complementing the results observed in the overexpression scenario (Figure 3C), cells with decreased Willin expression (Fibro-siWillin), displayed no statistically significant difference in the number of cells observed in G0/G1, S or G2/M phases compared to Fibro-siCtr cells (Figure 3D). However, the morphology of Fibro-siWillin cells was notably different: at low density, the Fibro-siWillin cells had elongated cell processes and a more pronounced flat spindle shape compared to Fibro-siCtr cells; after three days, Fibro-siWillin cells organized into a dense and close network of multiple interconnected processes protruding from the body of each cell (Figure 3E). This effect was accompanied by a statistically significant 3.5 fold increase in cellular proliferation compared to Fibro-siCtr cells (Figure 3F). These observations suggest that in fibroblasts Willin expression negatively regulates cellular proliferation but in a cell cycle independent manner.

### Willin Expression Promotes Fibroblast Migration

**Figure 4:**
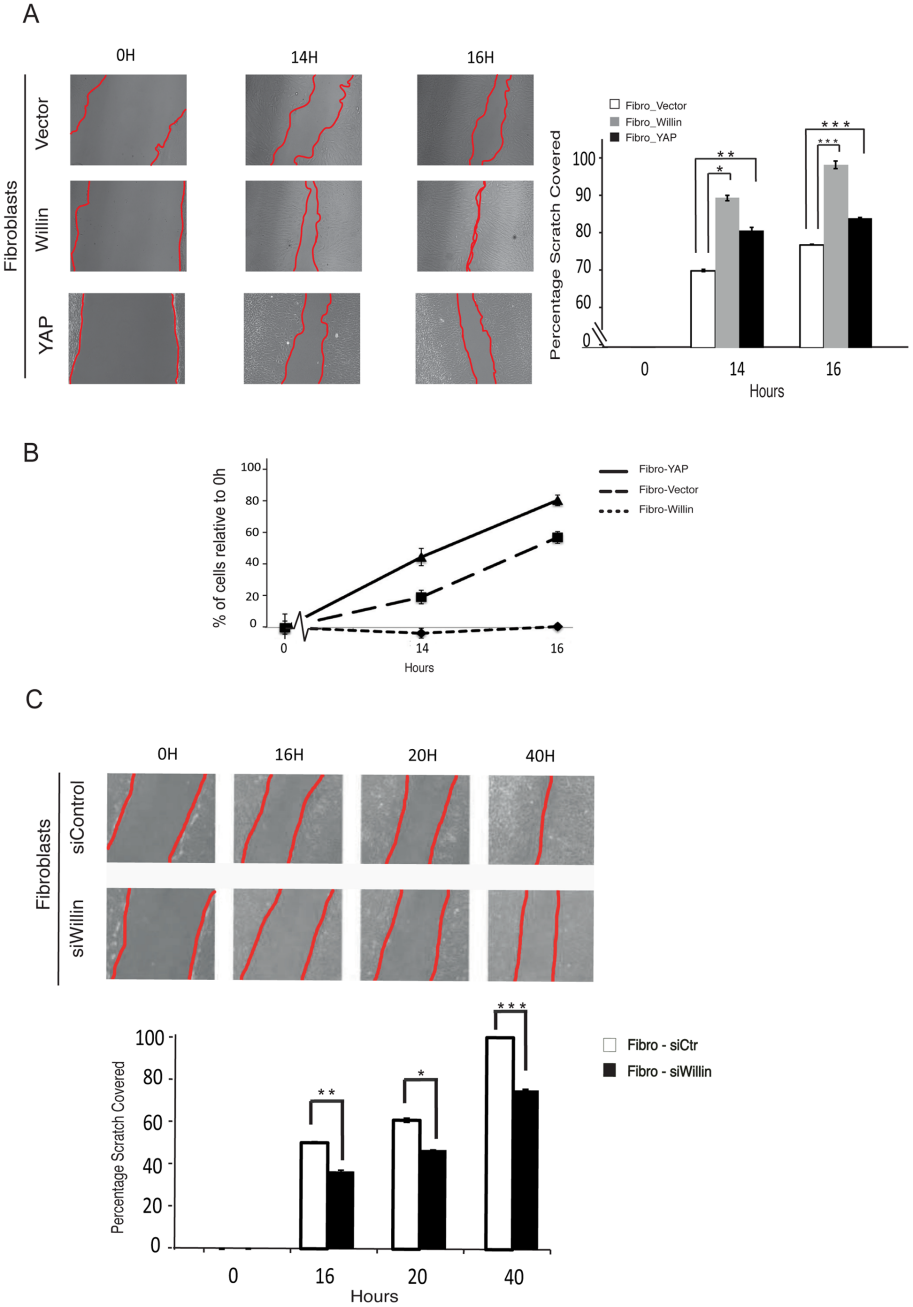
Willin expression promotes fibroblasts migration. Primary fibroblasts were retroviral infected with Willin, YAP or an empty vector. Stable pools were selected and maintained in normal fibroblasts medium. (A) Willin overexpression promotes faster cellular migration than YAP. Wound closure ability of fibroblasts stable cells was assessed by a wound-healing assay. Representative phase contrast images show the wounded area (0 h) and cell migration towards the wound after 14 h and 16 h. The percentage of scratch covered was measured by quantifying the total distance the cells moved from the edge of the scratch towards the center of the scratch (shown by red lines), using Image J software, followed by conversion to a percentage of scratch covered. Data is presented as the mean percentage of scratch covered in three independent experiments. Error bars represent ±s.d. (*n* = 3). (B) Willin promotion of wound closure is caused by directional cell migration and not by increased cell proliferation. The number of total cells in each one of the indicated time points was determined by cell count and the percentage of fibroblasts expressing vector, Willin or YAP relative to 0 h assessed. Means were calculated from three independent experiments. Error bars represent ±s.d. (*n* = 3). (C) Willin knockdown inhibits fibroblasts migration. Representative phase-contrast images of wound healing assay performed in fibro-siCtr or fibro-siWillin fibroblasts show the wounded area (0 h) and cell migration towards the wound after 16 h, 20 h and 40 h (shown by red lines). Motility was quantified as described. Data is presented as the mean percentage of scratch covered in three independent experiments. Error bars represent ±s.d. (*n* = 3).

Fibroblasts have an important role in wound healing, since these cells are the first to bridge the physical gap between the proximal and distal stumps following sciatic nerve transection [34], [35]. To assess the effect of Willin and YAP expression in promoting fibroblast wound closure, scratch assays were performed on confluent fibroblast monolayers and the rate of scratch closure was observed over 16 h. Strikingly, Fibro-Willin cells demonstrated a faster scratch closure rate compared to Fibro-Vector cells (Figure 4A). Cell numbers were counted to determine whether the faster scratch closure by Fibro-Willin cells was due to increased cellular proliferation or directional cell migration towards the wound. There was no change in the number of Fibro-Willin cells over the time course of the experiment in contrast to Fibro-YAP cells which showed increased cellular proliferation after 16 h compared to Fibro-Vector cells (Figure 4B). The impact of Willin on fibroblasts migration was further confirmed using Fibro-siWillin cells. As predicted, Fibro-siWillin cells displayed an inhibition of migration when compared to Fibro-siCtr cells (Figure 4C). Taken together, the results suggest that Willin expression promotes the migration of sciatic nerve derived fibroblasts.

### Willin is an Upstream Regulator of EphrinB2 and EGFR Expression

**Figure 5:**
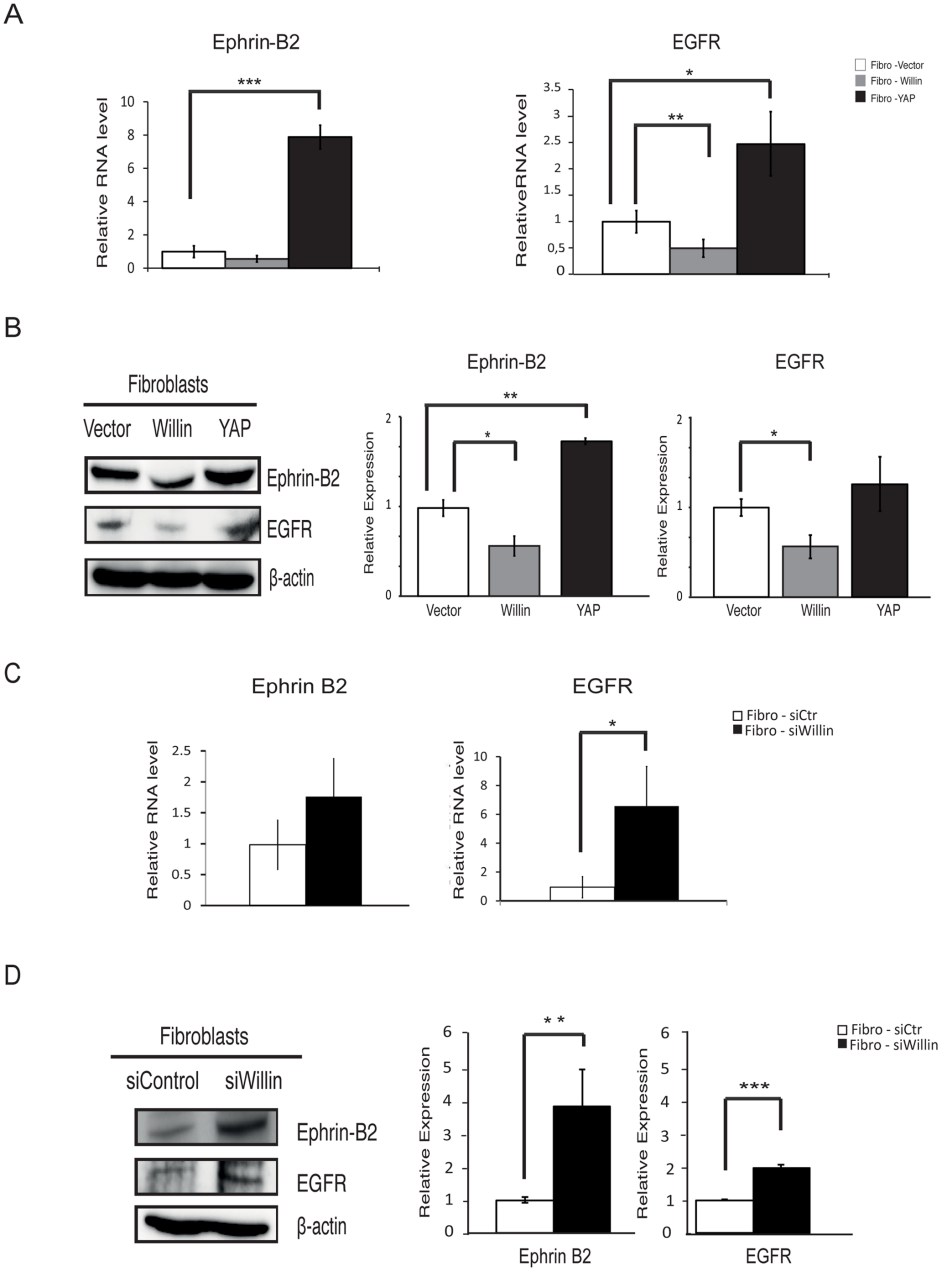
Willin is an upstream regulator of EphrinB2 and EGFR. (A) Willin expression is antagonistic to YAP EphrinB2 and EGFR transcriptional regulation. mRNA levels of Ephrin B2 and EGFR were probed by quantitative real-time PCR. mRNA levels were compared with the empty vector control fibroblast cells (set to 1). At the transcriptional level, Willin overexpression decreases EGFR and to a less extend EphrinB2 whereas YAP results in an increase in both EphrinB2 and EGFR mRNA levels. Means were calculated from *Ct* values in six independent experiments. β-actin was used to normalize for variances in input cDNA. Error bars represent ±s.e. (*n* = 6). Fibro-vector vs fibro-Willin or fibro-YAP for the analysed genes: *p<0.05; **p<0.01; ***p<0.001; Student’s *t*-test. Fibroblasts-vector vs fibroblasts-Willin: EphrinB2 (p = 0.055); Student’s *t*-test. (B) Immunoblots analysis of EphrinB2 and EGFR show loss of expression upon ectopic Willin expression and gain of EphrinB2 expression upon YAP overexpression when compared to empty vector control (set to 1). β-actin was used as a loading control. Means were calculated from six independent experiments. Error bars represent ±s.e. (*n* = 6). Fibro-vector vs fibro-Willin or fibro-YAP: *p<0.05; **p<0.01; Student’s *t*-test. Fibroblasts-vector vs fibroblasts-YAP: EGFR (p = 0.40); Student’s *t*-test. (C) EGFR mRNA levels are upregulated upon Willin knockdown but EphrinB2 does not show statistical significant change at the transcriptional level. mRNA levels of Ephrin B2 and EGFR were probed by quantitative real-time PCR and mRNA levels compared with the fibro-siCtr cells (set to 1). Means were calculated from *Ct* values in six independent experiments. β-actin was used to normalize for variances in input cDNA. Error bars represent ±s.e. (*n* = 6). Fibro-siCtr vs fibro-siWillin cells: *p<0.05; Student’s *t*-test. Fibro-siCtr vs Fibro-siWillin cells: EphrinB2 (p = 0.555); Student’s *t*-test. (D) Immunoblots analysis of EphrinB2 and EGFR show upregulated expression when Willin is knockdown. Relative expression was determined compared to background expression (Fibro-siCtr cells set to 1). β-actin was used as a loading control. Means were calculated from three independent experiments. Error bars represent ±s.e. (*n* = 3). Fibro-siCtr vs Fibro-siWillin cells: **p<0.01; ***p<0.001; Student’s *t*-test.

Previously, Parrinello and coworkers described ephrin/Eph signaling from fibroblasts is responsible for a directional axonal outgrowth of Schwann cells [26]. In order to establish whether the Hippo pathway plays a role in ephrin/Eph signaling, the expression of ephrinB2 was analyzed in Fibro-Willin, Fibro-YAP and Fibro-Vector cells. Quantitative RT-PCR (Figure 5A) and immunoblotting analysis (Figure 5B) showed that ectopic Willin expression resulted in ephrinB2 down-regulation whereas YAP induced ephrinB2 up-regulation as compared to Fibro-Vector cells. This finding was complemented by determining whether endogenous Willin regulates ephrinB2 expression. As predicted, knockdown of Willin in Fibro-siWillin cells resulted in an increase in ephrinB2 levels, both at mRNA (Figure 5C) and protein levels (Figure 5D), as compared to Fibro-siCtr cells.

**Figure 6:**
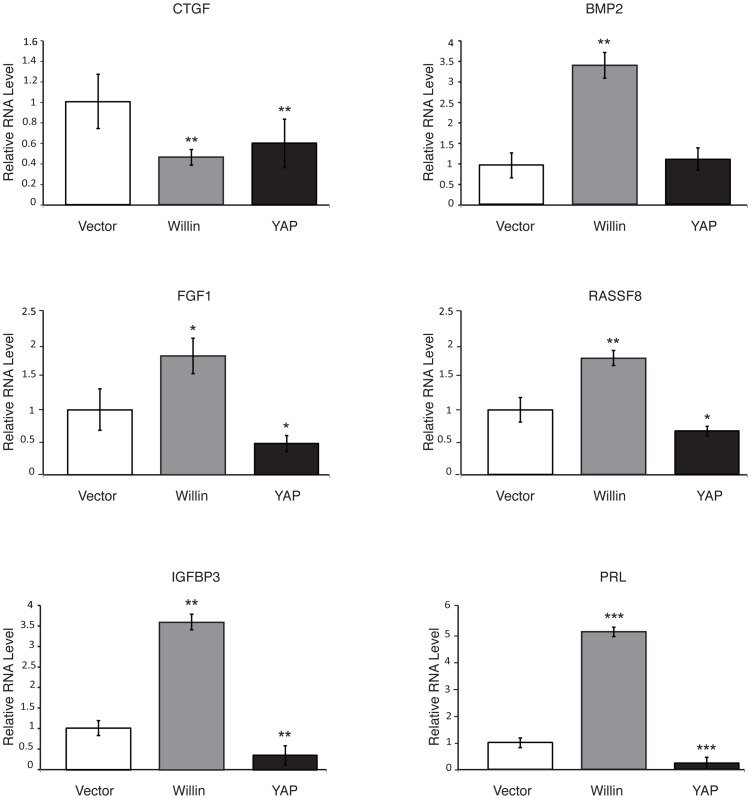
Willin antagonizes some of the genes regulated by YAP in sciatic nerve fibroblasts. mRNA expression of the YAP target genes CTGF, BMP2, FGF1, RASSF8, IGFBP3 and PRL was probed in fibroblasts expressing Willin or YAP by quantitative real-time PCR. mRNA levels were compared with the empty vector control (set to 1). Willin overexpression increased BMP2, FGF1, RASSF8, IGFBP3 and PRL mRNA levels and, together with YAP, decreased CTGF mRNA expression in these cells. Means were calculated from *Ct* values in three independent experiments. β-actin was used to normalize for variances in input cDNA. Error bars represent ±s.e. (*n* = 3). Fibroblasts-vector vs fibroblasts-Willin or fibroblasts-YAP for all the analysed genes: *p<0.05; **p<0.01; ***p<0.001; Student’s *t*-test. Fibroblasts-vector vs fibroblasts-YAP: BMP2 (p = 0.217); Student’s *t*-test.

These results were also closely mirrored when levels of the epidermal growth factor receptor (EGFR) were also monitored as previous studies have shown that activated EGFR leads to increased cell migration in wound repair mediated by fibroblasts [36]–[38]. Notably, there was a down-regulation of EGFR in Fibro-Willin cells compared to Fibro-Vector cells, both at the mRNA (Figure 5A) and protein levels (Figure 6B). Conversely, comparing the Fibro-YAP cells to the control Fibro-Vector cells resulted in a significant increase in EGFR mRNA levels (Figure 5A) but a non-significant increase at the protein level (Figure 5B). Confirming the role of Willin on EGFR expression, Fibro-siWillin cells displayed a predicted up-regulation of EGFR as compared to Fibro-siCtr cells, both at the mRNA (Figure 5C) and protein level (Figure 5D).

### Willin Influences Expression of some but not all Genes Regulated by YAP in Sciatic Nerve Fibroblasts

Previously, the Willin FERM domain was shown to be sufficient to influence the activity of genes regulated by YAP [9]. Since the Hippo kinase cascade has cell-specific regulatory functions in different tissues [4], [13] some of these genes were analyzed to determine whether they are influenced by Willin and YAP in sciatic nerve fibroblasts. To test this, quantitative RT-PCR analysis of CTGF, BMP2, FGF1, RASSF8, IGFBP3 and PRL mRNA levels was conducted on Fibro-Willin, Fibro-YAP and Fibro-Vector cells (Figure 6). PRL (prolactin), among all the analyzed genes was the most strongly up-regulated by 5-fold in Fibro-Willin cells when compared to Fibro-Vector cells, but down-regulated in Fibro-YAP cells. Intriguingly, CTGF, a direct YAP target gene in NIH-3T3 and MCF10A cells [39] was down-regulated in Fibro-YAP cells as compared to Fibro-Vector cells. Loss of CTGF expression was also observed in Fibro-Willin cells as compared to Fibro-Vector cells. Notably, FGF1 and IGFBP3 mRNA levels were significantly up-regulated in Fibro-Willin cells and down-regulated in Fibro-YAP cells, when compared to Fibro-Vector cells. Furthermore, BMP2 and RASSF8 were up-regulated in Fibro-Willin cells when compared to Fibro-Vector cells. These data demonstrate that, in fibroblasts isolated form the sciatic nerve, Willin and YAP have antagonistic regulatory functions upon FGF1, RASSF8, IGBP3 and PRL genes, but other genes show context-dependent regulation such that BMP2 expression is influenced by Willin and not YAP, while CTGF expression is inhibited by both Willin and YAP.

## Discussion

**Figure 7:**
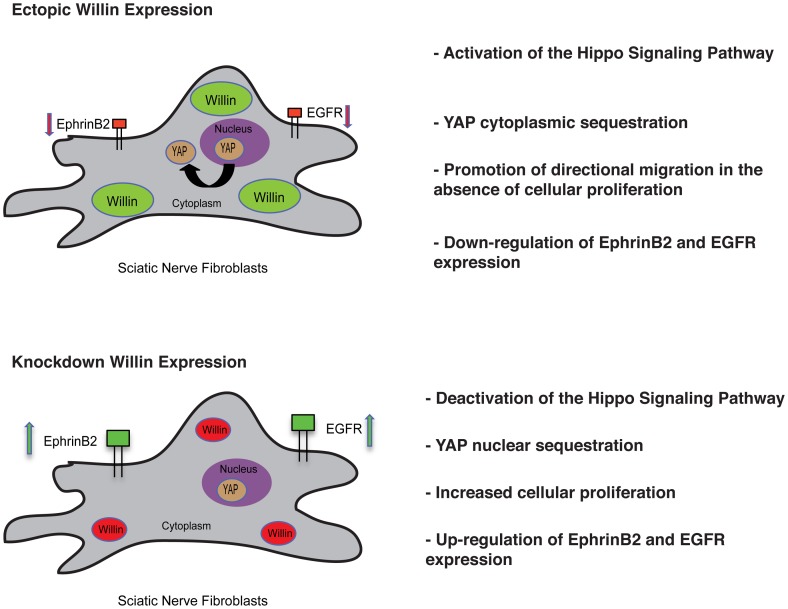
Willin orchestrates sciatic nerve fibroblasts. Ectopic expression of Willin activates the Hippo signaling pathway and YAP translocation from the nucleus to the cytoplasm. It also promotes wound closure by directional migration without increased cellular proliferation and induces down-regulation of ephrinB2 and EGFR expression. Knockdown of Willin expression further supports the aforementioned findings.

Although several advances have been made in the understanding of the emerging mammalian Hippo signaling pathway in its regulation of organ size control, tissue regeneration and stem-cell renewal [4], [13] little is known about the involvement of this pathway in the peripheral nervous system where Willin/FRMD6 was first identified [27]. This study establishes a role for Willin in mammalian peripheral nerve fibroblasts based on four findings. Firstly, we find that Willin as well as other components of the Hippo signaling pathway are expressed in these fibroblasts. Secondly, Willin has previously been shown to activate the Hippo pathway in epithelial cells [9], [11] and we extend this to fibroblasts, where Willin expression increases MST1/2, LATS1 and YAP phosphorylation, and consequently a shift of YAP from the nucleus to the cytoplasm. These findings were further confirmed by knockdown of Willin. Thirdly, we show that Willin expression promotes fibroblast wound closure by directional migration in the absence of proliferation. Fourthly, we show Willin expression down-regulates ephrinB2 and EGFR (Figure 7).

Fibroblasts are the main mediators of the dynamic and well-organized process of wound healing. They accumulate at the injury site [26], synthesize ECM components forming granulation tissue, and promote mechanical forces within the wound to initiate tissue contraction, a mechanism that leads to scar formation [40]. Fibroblasts are also involved in angiogenesis, promoting wound vascularization and inflammation by secreting proinflammatory cytokines [41]. If extensive invasion and proliferation of scar-forming fibroblasts occurs, pathophysiological conditions may arise such as neuromas or hypertrophic scars [42]. During the process of tissue repair, extracellular matrix goes through physical changes in terms of elasticity and cell shape. Recently YAP has been described as a crucial mediator of physical and mechanical cues in the cellular microenvironment, such that a “stiff” microenvironment activates YAP [43], [44]. Therefore, these modifications may exert mechanical signals that activate YAP, which would be antagonized by signals from the Hippo pathway.

Willin expression induces wound closure in the absence of proliferation and this might be associated with an initial injury event. When the microenvironment becomes more “stiff” upon the accumulation of fibroblasts, then YAP activation would predominate, increasing fibroblast proliferation, inhibiting fibroblast migration and causing increased Ephrin B2 and EGFR expression in the fibroblasts. Parrinello and coworkers (2010) [26] recently described that ephrinB2 ligand expression on fibroblasts induces activation of the EphB2 receptors located on Schwann cells. The ephrinB2/EphB2 signal results in an organized directional cell migration by the Schwann cells as it mediates their sorting in the form of multicellular cords to guide axons regrowth across the wound. Activation of EphB2 receptor on Schwann cells was found to be Sox2 dependent [26]. Sox2 is pivotal for the maintenance of pluripotency and regulation of stem cell self-renewal and differentiation [45], [46]. Interestingly, YAP has been shown to regulate Sox2 in mES cells [23] and we observed higher YAP expression in Schwann cells as compared to fibroblasts. Without an antagonizing signal this process would continue resulting in an excess of fibroblasts. In fact, ephrinB2 has been found overexpressed in different fibroproliferative diseases [47], [48]. Our data suggest a role for the Hippo pathway in the negative regulation of ephrinB2/EphB2 receptor signaling in fibroblasts, limiting excessive fibroblast proliferation and inappropriate Schwann cell activation by fibroblasts.

Several studies have shown that EGF, by activating EGFR, leads to increased cell migration, a feature of tumour progression, metastasis and wound healing [49], [50] but in other cell-types leads to increased cell proliferation [51], [52]. High EGFR expression has been shown in wound repair of both epithelial and human skin fibroblasts by promoting cell migration and wound epithelialization [37], [38], [53]. In our experiments, an increase in ectopic Willin expression induced down-regulation of EGFR, with an increase in cell migration but inhibition/delay of cellular proliferation, whilst a decrease in Willin expression resulted in an increase in EGFR expression with a inhibition/delay of cell migration but an increase in cell proliferation. This suggests that Willin expression can influence the level of EGFR in sciatic nerve fibroblasts, and that this in turn regulates the potential of over-proliferation, highlighting the fact that fibroblasts have different functions in a tissue-context dependent manner. In agreement with our observations, Merlin in mammalian cells also inhibits EGFR in mouse embryonic fibroblasts (MEFs) and cells undergo contact-dependent inhibition of proliferation [54]. Furthermore, in *Drosophila*, Merlin and Expanded mutant cells show an up-regulation of the EGFR signaling pathway [55]. It is also possible to hypothesise that there may also be an element of feedback control, as EGF itself has been shown to influence the cellular distribution of Willin [27]; and also, recently the EGFR ligand, amphiregulin, has been identified as a transcriptional YAP target [14].

Willin induced transcriptional activation of BMP2, FGF1, IGFBP3, PRL and RASSF8. Importantly, we observed that Willin and YAP displayed opposite effects on FGF1, IGFBP3, RASSF8 and PRL expression. Willin increased expression, while YAP decreased expression of these genes. While, BMP2 and RASSF8 displayed a similar trend in Willin-induced upregulation with the data presented by Moleirinho et al. (2013) and Angus et al. (2012) [9], [11], in epithelial MCF10A cells, FGF1, IGFBP3 and PRL were regulated by Willin expression but displayed the opposite trend. BMP2 has been shown to induce cell migration in different cell types, including MEF [56] and its involvement in the liver and peripheral nerve healing response has been previously described [57], [58]. The pronounced Willin-induced upregulation of BMP2 could also explain the migratory pattern observed in the wound-healing assay. Expression of CTGF, a YAP direct target [39], [59], in tissue wound repair has been proposed to be a major player in the pathogenesis of fibrotic processes [60]. We found it down regulated upon Willin expression.

Our data suggests that the hippo signaling pathway might have a significant role in both the development and maintenance of the mammalian peripheral nervous system. Specifically it would be of great interest to explore this further in both fibroblasts and Schwann cells to verify how the signaling cascade regulates the ephrin/Eph signaling, and its ability to control wound repair and, ultimately, regeneration of the PNS.

## References

[1] Edgar BA (2006) From cell structure to transcription: Hippo forges a new path. Cell 124: 267–273. 16439203 10.1016/j.cell.2006.01.005

[2] Harvey K, Tapon N (2007) The Salvador-Warts-Hippo pathway - an emerging tumour-suppressor network. Nature reviews Cancer 7: 182–191. 17318211 10.1038/nrc2070

[3] Pan D (2010) The hippo signaling pathway in development and cancer. Developmental cell 19: 491–505. 20951342 10.1016/j.devcel.2010.09.011 PMC3124840

[4] Zhao B, Tumaneng K, Guan KL (2011) The Hippo pathway in organ size control, tissue regeneration and stem cell self-renewal. Nature cell biology 13: 877–883. 21808241 10.1038/ncb2303 PMC3987945

[5] Dong J, Feldmann G, Huang J, Wu S, Zhang N, et al (2007) Elucidation of a universal size-control mechanism in Drosophila and mammals. Cell 130: 1120–1133. 17889654 10.1016/j.cell.2007.07.019 PMC2666353

[6] Oka T, Mazack V, Sudol M (2008) Mst2 and Lats kinases regulate apoptotic function of Yes kinase-associated protein (YAP). The Journal of biological chemistry 283: 27534–27546. 18640976 10.1074/jbc.M804380200

[7] Zhang J, Smolen GA, Haber DA (2008) Negative regulation of YAP by LATS1 underscores evolutionary conservation of the Drosophila Hippo pathway. Cancer research 68: 2789–2794. 18413746 10.1158/0008-5472.CAN-07-6205

[8] Zhao B, Wei X, Li W, Udan RS, Yang Q, et al (2007) Inactivation of YAP oncoprotein by the Hippo pathway is involved in cell contact inhibition and tissue growth control. Genes & development 21: 2747–2761. 17974916 10.1101/gad.1602907 PMC2045129

[9] Angus L, Moleirinho S, Herron L, Sinha A, Zhang X, et al (2012) Willin/FRMD6 expression activates the Hippo signaling pathway kinases in mammals and antagonizes oncogenic YAP. Oncogene 31: 238–250. 21666719 10.1038/onc.2011.224

[10] Camargo FD, Gokhale S, Johnnidis JB, Fu D, Bell GW, et al (2007) YAP1 increases organ size and expands undifferentiated progenitor cells. Current biology : CB 17: 2054–2060. 17980593 10.1016/j.cub.2007.10.039

[11] Moleirinho S, Chang N, Sims AH, Tilston-Lünel AM, Angus L, et al.. (2012) KIBRA exhibits MST-independent functional regulation of the Hippo signaling pathway in mammals. Oncogene. 10.1038/onc.2012.196 22614006

[12] Overholtzer M, Zhang J, Smolen GA, Muir B, Li W, et al (2006) Transforming properties of YAP, a candidate oncogene on the chromosome 11q22 amplicon. Proceedings of the National Academy of Sciences of the United States of America 103: 12405–12410. 16894141 10.1073/pnas.0605579103 PMC1533802

[13] Ramos A, Camargo FD (2012) The Hippo signaling pathway and stem cell biology. Trends in cell biology 22: 339–346. 22658639 10.1016/j.tcb.2012.04.006 PMC3383919

[14] Zhang J, Ji JY, Yu M, Overholtzer M, Smolen GA, et al (2009) YAP-dependent induction of amphiregulin identifies a non-cell-autonomous component of the Hippo pathway. Nature cell biology 11: 1444–1450. 19935651 10.1038/ncb1993 PMC2819909

[15] Zhou D, Conrad C, Xia F, Park JS, Payer B, et al (2009) Mst1 and Mst2 maintain hepatocyte quiescence and suppress hepatocellular carcinoma development through inactivation of the Yap1 oncogene. Cancer cell 16: 425–438. 19878874 10.1016/j.ccr.2009.09.026 PMC3023165

[16] Hamaratoglu F, Willecke M, Kango-Singh M, Nolo R, Hyun E, et al (2006) The tumour-suppressor genes NF2/Merlin and Expanded act through Hippo signalling to regulate cell proliferation and apoptosis. Nature cell biology 8: 27–36. 16341207 10.1038/ncb1339

[17] Xiao L, Chen Y, Ji M, Dong J (2011) KIBRA regulates Hippo signaling activity via interactions with large tumor suppressor kinases. The Journal of biological chemistry 286: 7788–7796. 21233212 10.1074/jbc.M110.173468 PMC3048666

[18] Yu J, Zheng Y, Dong J, Klusza S, Deng WM, et al (2010) Kibra functions as a tumor suppressor protein that regulates Hippo signaling in conjunction with Merlin and Expanded. Developmental cell 18: 288–299. 20159598 10.1016/j.devcel.2009.12.012 PMC2858562

[19] Zhang N, Bai H, David KK, Dong J, Zheng Y, et al (2010) The Merlin/NF2 tumor suppressor functions through the YAP oncoprotein to regulate tissue homeostasis in mammals. Developmental cell 19: 27–38. 20643348 10.1016/j.devcel.2010.06.015 PMC2925178

[20] Judson RN, Tremblay AM, Knopp P, White RB, Urcia R, et al.. (2012) The Hippo pathway member Yap plays a key role in influencing fate decisions in muscle satellite cells. Journal of cell science. 10.1242/jcs.109546 PMC3585517 23038772

[21] Charge SB, Rudnicki MA (2004) Cellular and molecular regulation of muscle regeneration. Physiological reviews 84: 209–238. 14715915 10.1152/physrev.00019.2003

[22] Cai J, Zhang N, Zheng Y, de Wilde RF, Maitra A, et al (2010) The Hippo signaling pathway restricts the oncogenic potential of an intestinal regeneration program. Genes & development 24: 2383–2388. 21041407 10.1101/gad.1978810 PMC2964748

[23] Lian I, Kim J, Okazawa H, Zhao J, Zhao B, et al (2010) The role of YAP transcription coactivator in regulating stem cell self-renewal and differentiation. Genes & development 24: 1106–1118. 20516196 10.1101/gad.1903310 PMC2878649

[24] Harrisingh MC, Perez-Nadales E, Parkinson DB, Malcolm DS, Mudge AW, et al (2004) The Ras/Raf/ERK signalling pathway drives Schwann cell dedifferentiation. The EMBO journal 23: 3061–3071. 15241478 10.1038/sj.emboj.7600309 PMC514926

[25] Napoli I, Noon LA, Ribeiro S, Kerai AP, Parrinello S, et al (2012) A central role for the ERK-signaling pathway in controlling Schwann cell plasticity and peripheral nerve regeneration in vivo. Neuron 73: 729–742. 22365547 10.1016/j.neuron.2011.11.031

[26] Parrinello S, Napoli I, Ribeiro S, Digby PW, Fedorova M, et al (2010) EphB signaling directs peripheral nerve regeneration through Sox2-dependent Schwann cell sorting. Cell 143: 145–155. 20869108 10.1016/j.cell.2010.08.039 PMC3826531

[27] Gunn-Moore FJ, Welsh GI, Herron LR, Brannigan F, Venkateswarlu K, et al (2005) A novel 4.1 ezrin radixin moesin (FERM)-containing protein, ‘Willin’. FEBS letters 579: 5089–5094. 16137681 10.1016/j.febslet.2005.07.097

[28] Boedigheimer M, Laughon A (1993) Expanded: a gene involved in the control of cell proliferation in imaginal discs. Development 118: 1291–1301. 8269855 10.1242/dev.118.4.1291

[29] Brockes JP, Fields KL, Raff MC (1979) Studies on cultured rat Schwann cells. I. Establishment of purified populations from cultures of peripheral nerve. Brain research 165: 105–118. 371755 10.1016/0006-8993(79)90048-9

[30] Lakatos A, Franklin RJ, Barnett SC (2000) Olfactory ensheathing cells and Schwann cells differ in their in vitro interactions with astrocytes. Glia 32: 214–225. 11102963 10.1002/1098-1136(200012)32:3<214::aid-glia20>3.0.co;2-7

[31] Tait S, Gunn-Moore F, Collinson JM, Huang J, Lubetzki C, et al (2000) An oligodendrocyte cell adhesion molecule at the site of assembly of the paranodal axo-glial junction. The Journal of cell biology 150: 657–666. 10931875 10.1083/jcb.150.3.657 PMC2175192

[32] Davis JQ, Lambert S, Bennett V (1996) Molecular composition of the node of Ranvier: identification of ankyrin-binding cell adhesion molecules neurofascin (mucin+/third FNIII domain-) and NrCAM at nodal axon segments. The Journal of cell biology 135: 1355–1367. 8947556 10.1083/jcb.135.5.1355 PMC2121080

[33] Gunn-Moore FJ, Hill M, Davey F, Herron LR, Tait S, et al (2006) A functional FERM domain binding motif in neurofascin. Molecular and cellular neurosciences 33: 441–446. 17045809 10.1016/j.mcn.2006.09.003

[34] Scaravilli F (1984) Regeneration of the perineurium across a surgically induced gap in a nerve encased in a plastic tube. Journal of anatomy 139 (Pt 3): 411–424. PMC1165057 6490525

[35] Schroder JM, May R, Weis J (1993) Perineurial cells are the first to traverse gaps of peripheral nerves in silicone tubes. Clinical neurology and neurosurgery 95 Suppl: S78–83 10.1016/0303-8467(93)90040-n 8467601

[36] Brown GL, Nanney LB, Griffen J, Cramer AB, Yancey JM, et al (1989) Enhancement of wound healing by topical treatment with epidermal growth factor. The New England journal of medicine 321: 76–79. 2659995 10.1056/NEJM198907133210203

[37] Cao C, Sun Y, Healey S, Bi Z, Hu G, et al (2006) EGFR-mediated expression of aquaporin-3 is involved in human skin fibroblast migration. The Biochemical journal 400: 225–234. 16848764 10.1042/BJ20060816 PMC1652825

[38] Carpenter G, Cohen S (1990) Epidermal growth factor. The Journal of biological chemistry 265: 7709–7712. 2186024

[39] Zhao B, Ye X, Yu J, Li L, Li W, et al (2008) TEAD mediates YAP-dependent gene induction and growth control. Genes & development 22: 1962–1971. 18579750 10.1101/gad.1664408 PMC2492741

[40] Gabbiani G, Hirschel BJ, Ryan GB, Statkov PR, Majno G (1972) Granulation tissue as a contractile organ. A study of structure and function. The Journal of experimental medicine 135: 719–734. 4336123 10.1084/jem.135.4.719 PMC2139162

[41] Sorrell JM, Caplan AI (2009) Fibroblasts-a diverse population at the center of it all. International review of cell and molecular biology 276: 161–214. 19584013 10.1016/S1937-6448(09)76004-6

[42] Burnett MG, Zager EL (2004) Pathophysiology of peripheral nerve injury: a brief review. Neurosurgical focus 16: E1. 10.3171/foc.2004.16.5.2 15174821

[43] Dupont S, Morsut L, Aragona M, Enzo E, Giulitti S, et al (2011) Role of YAP/TAZ in mechanotransduction. Nature 474: 179–183. 21654799 10.1038/nature10137

[44] Halder G, Dupont S, Piccolo S (2012) Transduction of mechanical and cytoskeletal cues by YAP and TAZ. Nature reviews Molecular cell biology 13: 591–600. 10.1038/nrm3416 22895435

[45] Chambers I, Tomlinson SR (2009) The transcriptional foundation of pluripotency. Development 136: 2311–2322. 19542351 10.1242/dev.024398 PMC2729344

[46] Takahashi K, Yamanaka S (2006) Induction of pluripotent stem cells from mouse embryonic and adult fibroblast cultures by defined factors. Cell 126: 663–676. 16904174 10.1016/j.cell.2006.07.024

[47] Umeda N, Ozaki H, Hayashi H, Oshima K (2004) Expression of ephrinB2 and its receptors on fibroproliferative membranes in ocular angiogenic diseases. American journal of ophthalmology 138: 270–279. 15289137 10.1016/j.ajo.2004.04.006

[48] Wynn TA (2007) Common and unique mechanisms regulate fibrosis in various fibroproliferative diseases. The Journal of clinical investigation 117: 524–529. 17332879 10.1172/JCI31487 PMC1804380

[49] Fujii K, Dousaka-Nakajima N, Imamura S (1995) Epidermal growth factor enhancement of HSC-1 human cutaneous squamous carcinoma cell adhesion and migration on type I collagen involves selective up-regulation of alpha 2 beta 1 integrin expression. Experimental cell research 216: 261–272. 7529189 10.1006/excr.1995.1032

[50] Matthay MA, Thiery JP, Lafont F, Stampfer F, Boyer B (1993) Transient effect of epidermal growth factor on the motility of an immortalized mammary epithelial cell line. Journal of cell science 106 (Pt 3): 869–878. 10.1242/jcs.106.3.869 8308069

[51] Traverse S, Gomez N, Paterson H, Marshall C, Cohen P (1992) Sustained activation of the mitogen-activated protein (MAP) kinase cascade may be required for differentiation of PC12 cells. Comparison of the effects of nerve growth factor and epidermal growth factor. The Biochemical journal 288 (Pt 2): 351–355. 10.1042/bj2880351 PMC1132018 1334404

[52] Xie Z, Jiang Y, Liao EY, Chen Y, Pennypacker SD, et al (2012) PIKE mediates EGFR proliferative signaling in squamous cell carcinoma cells. Oncogene 31: 5090–5098. 22349826 10.1038/onc.2012.10

[53] Block ER, Klarlund JK (2008) Wounding sheets of epithelial cells activates the epidermal growth factor receptor through distinct short- and long-range mechanisms. Molecular biology of the cell 19: 4909–4917. 18799627 10.1091/mbc.E08-01-0097 PMC2575185

[54] Curto M, Cole BK, Lallemand D, Liu CH, McClatchey AI (2007) Contact-dependent inhibition of EGFR signaling by Nf2/Merlin. The Journal of cell biology 177: 893–903. 17548515 10.1083/jcb.200703010 PMC2064288

[55] Maitra S, Kulikauskas RM, Gavilan H, Fehon RG (2006) The tumor suppressors Merlin and Expanded function cooperatively to modulate receptor endocytosis and signaling. Current biology : CB 16: 702–709. 16581517 10.1016/j.cub.2006.02.063

[56] Gamell C, Susperregui AG, Bernard O, Rosa JL, Ventura F (2011) The p38/MK2/Hsp25 pathway is required for BMP-2-induced cell migration. PloS one 6: e16477. 21297993 10.1371/journal.pone.0016477 PMC3030584

[57] Oumi N, Taniguchi KA, Kanai AM, Yasunaga M, Nakanishi T, et al (2012) A crucial role of bone morphogenetic protein signaling in the wound healing response in acute liver injury induced by carbon tetrachloride. International journal of hepatology 2012: 476820. 22701178 10.1155/2012/476820 PMC3372049

[58] Tsujii M, Akeda K, Iino T, Uchida A (2009) Are BMPs involved in normal nerve and following transection?: a pilot study. Clinical orthopaedics and related research 467: 3183–3189. 19669850 10.1007/s11999-009-1009-1 PMC2772907

[59] Leask A, Abraham DJ (2006) All in the CCN family: essential matricellular signaling modulators emerge from the bunker. Journal of cell science 119: 4803–4810. 17130294 10.1242/jcs.03270

[60] Brigstock DR (1999) The connective tissue growth factor/cysteine-rich 61/nephroblastoma overexpressed (CCN) family. Endocrine reviews 20: 189–206. 10204117 10.1210/edrv.20.2.0360

